# Positive selection drives adaptive diversification of the 4-coumarate: CoA ligase (*4CL*) gene in angiosperms

**DOI:** 10.1002/ece3.1613

**Published:** 2015-07-23

**Authors:** Haiyan Sun, Kai Guo, Shengqiu Feng, Weihua Zou, Ying Li, Chunfen Fan, Liangcai Peng

**Affiliations:** 1 School of Biology and Food Engineering, Changshu Institute of TechnologyChangshu, 215500, China; 2 National Key Laboratory of Crop Genetic Improvement, Huazhong Agricultural UniversityWuhan, 430070, China; 3 Biomass and Bioenergy Research Centre, Huazhong Agricultural UniversityWuhan, 430070, China; 4 College of Life Science and Technology, Huazhong Agricultural UniversityWuhan, 430070, China; 5 College of Plant Science and Technology, Huazhong Agricultural UniversityWuhan, 430070, China

**Keywords:** 4-Coumarate: coenzyme A ligase, diversification, evolution, phylogeny, positive selection

## Abstract

Lignin and flavonoids play a vital role in the adaption of plants to a terrestrial environment. 4-Coumarate: coenzyme A ligase (4CL) is a key enzyme of general phenylpropanoid metabolism which provides the precursors for both lignin and flavonoids biosynthesis. However, very little is known about how such essential enzymatic functions evolve and diversify. Here, we analyze 4CL sequence variation patterns in a phylogenetic framework to further identify the evolutionary forces that lead to functional divergence. The results reveal that lignin-biosynthetic 4CLs are under positive selection. The majority of the positively selected sites are located in the substrate-binding pocket and the catalytic center, indicating that nonsynonymous substitutions might contribute to the functional evolution of 4CLs for lignin biosynthesis. The evolution of 4CLs involved in flavonoid biosynthesis is constrained by purifying selection and maintains the ancestral role of the protein in response to biotic and abiotic factors. Overall, our results demonstrate that protein sequence evolution via positive selection is an important evolutionary force driving adaptive diversification in 4CL proteins in angiosperms. This diversification is associated with adaption to a terrestrial environment.

## Introduction

Lignin and flavonoids are thought to play vital roles in the adaptation of plants to terrestrial environments (Rozemaa et al. 2002; Weng and Chapple 2010; Agati et al. 2013). The enzyme 4-Coumarate: CoA ligase (4CL; EC 6.2.1.12) is a key enzyme that functions in an early step of the general phenylpropanoid pathway. The protein 4CL converts 4-coumaric acid and other cinnamic acids, such as caffeic acid and ferulic acid, into the corresponding CoA thiol esters, which are then subsequently used for the biosynthesis of numerous secondary metabolites, including flavonoids, isoflavonoids, lignin, suberins, coumarins and wall-bound phenolics (Ehlting et al. 1999; Saballos et al. 2012). The *4CL* gene family is typically small. The *4CL* family has 4 members in *Arabidopsis* (Hamberger and Hahlbrock 2004), 5 members in rice (Gui et al. 2011; Sun et al. 2013), and 4 members in soybean (Lindermayr et al. 2002). 4CL isoforms with different substrate specificities may direct the flow from general phenylpropanoid metabolism into the different pathways for specific end products (Souza et al. 2008).

In dicots, 4CLs can be divided into two distinct groups: class I and class II. The disruption of 4CL expression has demonstrated that class I 4CLs participate in lignin formation, while class II 4CLs impact flavonoid metabolism (Lee et al. 1997; Hu et al. 1998; Ehlting et al. 1999; Harding et al. 2002; Nakashima et al. 2008). The remarkable functional diversity of 4CL suggests that it may be subject to positive Darwinian selection. However, how the *4CL* genes evolve and functionally diverge and whether natural selection plays a role in their evolution have been poorly studied. In this study, we analyzed nucleotide divergence in the *4CL* genes from 16 species and used likelihood methods with various evolutionary models to investigate potential patterns of positive selection.

## Methods

### Sequence data collection

All known and reported 4CL protein-coding sequences from dicots, monocots, and gymnosperms (loblolly pine) were retrieved from the National Center for Biotechnology Information (NCBI). In total, 42 4CL protein sequences from 16 species were collected and are listed in Table S1.

### Phylogenetic analysis

The 4CL protein-coding sequences were aligned using the program CLUSTALW implemented in MEGA5 (Tamura et al. 2011) and manually edited. Highly variable regions, indels, and gaps were excluded. A phylogenetic tree was constructed using MEGA5 with the neighbor-joining (NJ) method. The reliability of the branches was evaluated by 1000 bootstrap replicates.

### Test for selection

The nonsynonymous–synonymous substitution rate ratio (*ω* = dN/dS) provides a measure of the selective pressure at the protein level, where a *ω* of 1, <1, or >1 indicates neutral evolution, purifying selection, or positive selection, respectively. The hypothesis of positive selection was tested using the CODEML program in the PAML v4.3b package (Yang 2007). Three approaches, branch, site, and branch-site models, incorporated into the program were used. In the lineage-specific selection analyses, we employed the recently developed dynamic programming procedure to search for the optimal branch-specific model that had a likelihood equal to or close to the global maximum likelihood for all of the possible models (Zhang et al. 2011). In the site-specific selection analyses, the dataset was fitted to three pairs of codon substitution models (M2a vs. M1a, M3 vs. M0, and M8 vs. M7). The branch-site model A was used to detect positively selected sites along the branches that showed elevated *ω* ratios. The sites under positive selection were identified by the Bayes Empirical Bayes (BEB) approach.

## Results and Discussion

### Angiosperm 4CL gene phylogeny

**Figure 1:**
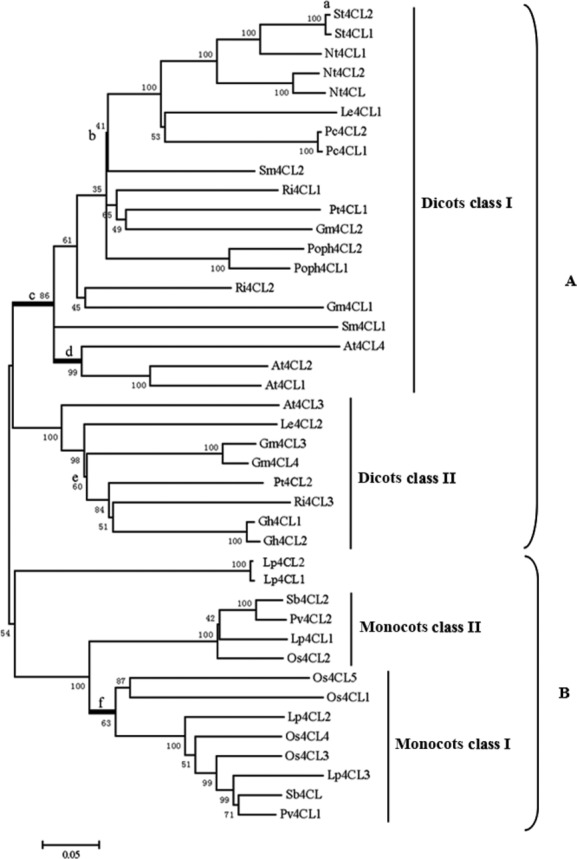
Phylogenetic relationship between 4CL genes from angiosperms based on the neighbor-joining method. The branch lengths are proportional to distances, and the values at the interior nodes are the bootstrap percentages derived from 1000 replicates. The six branches potentially under positive selection are indicated as a, b, c, d, e, and f, respectively.

The conserved protein-coding sequences of 42 4CLs from 16 species were used to reconstruct a phylogenetic tree. Analysis revealed that all of the *4CL* genes fell into one of two general groups: A and B (Fig. 1). Group A contains representatives from all of the available dicots, including verified 4CL sequences from Arabidopsis, poplar and soybean. The monocot 4CL isoenzymes in group B form a highly supported monophyletic group and are thus separated from the dicot isoforms. The gymnosperm 4CLs, the loblolly pine isoforms Lp4CL1 and Lp4CL2, also formed a separate cluster that was closest to the monocot isoenzymes.

### The functional divergence of the *4CL* gene family

The dicot *4CLs* can be divided into two distinct groups that are designated dicots class I and dicots class II (Fig. 1). Previous studies have demonstrated that *4CL* genes in dicots class I are associated with lignin accumulation, while dicots class II *4CLs* are involved in the metabolism of other phenolic compounds, such as flavonoids. For example, the genes *Pt4CL1*, *At4CL1*, *At4CL2*, *At4CL4*, *Gm4CL1,* and *Gm4CL2* in dicots class I are involved in lignin formation (Hu et al. 1998; Ehlting et al. 1999; Lindermayr et al. 2002). However, the genes *Pt4CL2*, *At4CL3,* and *Gm4CL4* in dicots class II are believed to play a role in flavonoid biosynthesis (Uhlmann and Ebel 1993; Hu et al. 1998; Ehlting et al. 1999).

The *4CLs* from monocots can also be classified into two groups, which are designated monocots class I and monocots class II (Fig. 1). The *4CL* genes in monocots class I are associated with lignin accumulation. For example, *Pv4CL1* in monocots class I is the key 4CL isoenzyme involved in lignin biosynthesis because RNA interference of *Pv4CL1* reduces the activity of extractable 4CL by 80% leading to a reduction in lignin content and a decrease in the guaiacyl unit composition (Xu et al. 2011). The *Os4CL3* gene in the same group is also involved in lignin biosynthesis because suppression of *Os4CL3* expression results in significant lignin reduction, retarded growth and other morphological changes (Gui et al. 2011). However, the genes in monocots class II (Fig. 1) are likely to participate in the flavonoid biosynthetic pathway. For example, based on phylogenetic analysis, Xu et al. (2011) hypothesized that *Pv4CL2* in monocots class II mainly participates in the flavonoid biosynthesis pathway in switchgrass. Recent research (Sun et al. 2013) has demonstrated that the primary function of *Os4CL2* is to channel the activated 4-coumarate to chalcone synthase and subsequently to different branched pathways of flavonoid secondary metabolism leading to flower pigments and UV protective flavonols and anthocyanins. The remarkable functional diversity of not only dicot but also monocot *4CLs* suggests that *4CL* may be subject to positive Darwinian selection.

### Evolutionary patterns among lineages and among sites

To test the hypothesis that positive selection acts on 4CLs, we applied branch-specific models to the 4CL dataset. It was clear that 40RM (40 ratio model) with 40 different *ω* ratios was the optimal branch model (Table S2). The six branches where *ω* was >1 were defined as branches *a*, *b*, *c*, *d*, *e,* and *f*, respectively (Fig. 1). To examine whether the *ω* ratio for each branch was significantly greater than the background ratio, the log-likelihood values were calculated from two-ratio models that assigned the ratios *ω*_*a*_, *ω*_*b*_, *ω*_*c*_, *ω*_*d*_, *ω*_*e*_, and *ω*_*f*_ to branches *a*, *b*, *c*, *d*, *e,* and *f*, and the ratio *ω*_0_ was assigned to all other branches. All of these two-ratio models were individually compared with the one-ratio model (M0). The one-ratio model, which assumes the same *ω* parameter for the entire tree, yielded a log-likelihood value of -28951.48 with an estimated *ω*_0_ of 0.089 (Table 1). The low average ratio indicated the dominating role of purifying selection in the evolution of the *4CL* genes. The two-ratio models for branches *a*, *c*, *d,* and *f* fit the data significantly better than the one-ratio model (Table 2), resulting in the rejection of the null hypothesis that the *4CL* genes evolved at constant rates along the branches. To test whether the six *ω* ratios were significantly higher than 1, we calculated the log-likelihood values using the two-ratio models with *ω*_*a*_, *ω*_*b*_, *ω*_*c*_, *ω*_*d*_, *ω*_*e*_, and *ω*_*f*_ fixed to 1 (Table 1). The likelihood ratio tests were also implemented for comparing each two-ratio model and its corresponding fixed two-ratio model. The likelihood ratio tests in Table 2 revealed that the *ω* ratios for branches *a*, *b*, *c*, *d*, *e,* and *f* were not significantly greater than one. We therefore conclude that the evolution of the *4CL* genes in angiosperms is dominated by purifying selection.

**Table 1:** Log-likelihood values and parameter estimates for the 4CL genes

Model	p	lnL	Parameter estimates	Positively selected sites
M0: one ratio	1	−28951.48	*ω*_0_ = 0.089	None
Branch specific models				
Two ratios (branch a)	2	−28949.50	*ω*_0_ = 0.089, *ω*_*a*_ = 3.463	
Two ratios (fixed *ω*_*a*_ = 1)	1	−28949.58	*ω*_0_ = 0.089, *ω*_*a*_ = 1	
Two ratios (branch b)	2	−28950.10	*ω*_0_ = 0.088, *ω*_*b*_ = 0.273	
Two ratios (fix *ω*_*b*_ = 1)	1	−28950.49	*ω*_0_ = 0.088, *ω*_*b*_ = 1	
Two ratios (branch c)	2	−28948.26	*ω*_0_ = 0.088, *ω*_*c*_ = 2.270	
Two ratios (fixed *ω*_*c*_ = 1)	1	−28948.29	*ω*_0_ = 0.088, *ω*_*c*_ = 1	
Two ratios (branch d)	2	−28948.76	*ω*_0_ = 0.088, *ω*_*d*_ = 0.574	
Two ratios (fixed *ω*_*d*_ = 1)	1	−28948.79	*ω*_0_ = 0.088, *ω*_*d*_ = 1	
Two ratios (branch e)	2	−28950.04	*ω*_0_ = 0.088, *ω*_*e*_ = ∞	
Two ratios (fixed *ω*_*e*_ = 1)	1	−28950.17	*ω*_0_ = 0.088, *ω*_*e*_ = 1	
Two ratios (branch f)	2	−28939.38	*ω*_0_ = 0.088, *ω*_*f*_ = ∞	
Two ratios (fixed *ω*_*f*_ = 1)	1	−28940.20	*ω*_0_ = 0.088, *ω*_*f*_ = 1	
Sites-specific models				
M1:neutral (K = 2)	1	−28731.92	*p*_0_ = 0.946 (*p*_1_ = 0.054)	Not allowed
M2: selection (K = 3)	3	−28731.92	*p*_0_ = 0.946, *p*_1_ = 0.011	None
			(*p*_2_ = 0.042), *ω*_2_ = 1	
M3: discrete (K = 2)	3	−28260.60	*p*_0_ = 0.520 (*p*_1_ = 0.480)	None
			*ω*_0_ = 0.020, *ω*_1_ = 0.175	
M3: discrete (K = 3)	5	−28136.31	*p*_0_ = 0.408, *p*_1_ = 0.456, (*p*_2_ = 0.136)	None
			*ω*_0_ = 0.010, *ω*_1_ = 0.108, *ω*_2_ = 0.320	
M7: beta	2	−28118.00	*p* = 0.542, *q* = 4.443	Not allowed
M8: beta and *ω*	4	−28116.60	*p*_0_ = 0.994, *p* = 0.570, *q* = 5.015 (*p*_1_ = 0.006), *ω* = 1	None
Branch-site model A				
Model a	3	−28731.43	*p*_0_ = 0.912, *p*_1_ = 0.052	None
			(*p*_2_ + *p*_3_ = 0.036), *ω*_2_ = 9.870	
Model fixed *ω*_*a*_	2	−28731.55	*p*_0_ = 0.725, *p*_1_ = 0.041	Not allowed
			(*p*_2_ + *p*_3_ = 0.234), *ω*_2_ = 1	
Model c	3	−28719.14	*p*_0_ = 0.885, *p*_1_ = 0.051	82C 291S (at *P* > 0.95)
			(*p*_2_ + *p*_3_ = 0.064), *ω*_2_ = 15.947	379M 423T (at *P* > 0.99)
Model fixed *ω*_*c*_	2	−28724.93	*p*_0_ = 0.780, *p*_1_ = 0.046	Not allowed
			(*p*_2_ + *p*_3_ = 0.155), *ω*_2_ = 1	
Model d	3	−28723.32	*p*_0_ = 0.922, *p*_1_ = 0.053	79V 202S 211S (at *P* > 0.95)
			(*p*_2_ + *p*_3_ = 0.024), *ω*_2_ = 14.873	
Model fixed *ω*_*d*_	2	−28726.61	*p*_0_ = 0.825, *p*_1_ = 0.047	Not allowed
			(*p*_2_ + *p*_3_ = 0.128), *ω*_2_ = 1	
Model f	3	−28716.01	*p*_0_ = 0.780, *p*_1_ = 0.045	65L 69E 181I 223L
			(*p*_2_ + *p*_3_ = 0.160), *ω*_2_ = ∞	234K 239K (at *P* > 0.95)
Model fixed *ω*_*f*_	2	−28720.97	*p*_0_ = 0.349, *p*_1_ = 0.020	Not allowed
			(*p*_2_ + *p*_3_ = 0.631), *ω*_2_ = 1	

**Table 2:** Likelihood ratio statistics (2ΔlnL) for testing branch hypothesis

	M0 (one ratio)	Fixed *ω*_*a*_ = 1	Fixed *ω*_*b*_ = 1	Fixed *ω*_*c*_ = 1	Fixed *ω*_*d*_ = 1	Fixed *ω*_*e*_ = 1	Fixed *ω*_*f*_ = 1
*ω*_*a*_ free	3.96*	0.16					
*ω*_*b*_ free	2.76		0.78				
*ω*_*c*_ free	6.44*			0.06			
*ω*_*d*_ free	5.44*				0.06		
*ω*_*e*_ free	2.88					0.26	
*ω*_*f*_ free	24.2**						1.64

* Significant (*P* < 0.05, *χ*^2^ = 3.84).

** Extremely significant (*P* < 0.01, *χ*^2^ = 6.63).

Because the branch model test averages the *ω* ratios across all of the sites and is a very conservative test for positive selection, we applied site-specific models to the 4CL dataset. The log-likelihood values and the parameter estimates under models with variable *ω* ratios among the sites are listed in Table 1. Two site classes (M3, K = 2) fit the data significantly better than one site class (M0) by 690.88 log-likelihood units revealing significant variation in the selective pressure on the sites. However, none of the site-specific models allowed for the presence of positively selected sites, such as M2a (selection), M3 (discrete), and M8 (beta and *ω*), suggesting the existence of positively selected sites with *ω* > 1. The majority of the sites in the 4CL sequences appear to be under strong selective constraints.

### Evidence for positive selection on lignin-related 4CL genes

Positive selection is difficult to detect because it often operates episodically on just a few amino acid sites and purifying selection may mask the signal. Branch-site models can detect positive selection that affected a small number of sites along prespecified lineages. We used branch-site model A to test the hypothesis. As detailed in Table 3, branch-site model A using branch *c* as the foreground branch (MAc) resulted in a significantly better fit to M1a (2ΔlnL = 25.56, df = 2, *P* < 0.00) and to null model A for branch *c* (2ΔlnL = 11.58, df = 1, *P* < 0.00) (Table 3). This result also suggested that 5.1% of amino acids are under positive selection in lineage c with *ω* = 15.95 (Table 1). Branch-site model A using branch *d* as the foreground branch (MAd) provided a significantly better fit to M1a (2ΔlnL = 17.2, df = 2, *P* < 0.00) and the null model A for branch *d* (2ΔlnL = 6.58, df = 1, *P* < 0.00) (Table 3). This result also suggested that 5.3% of the protein sites are under positive selection in lineage *d* with *ω* = 14.873 (Table 1). When the analysis was repeated with branch *f* as the foreground branch (MAf), model A was much more realistic and fit the data significantly better than M1a (2ΔlnL = 31.82, df = 2, *P* < 0.00) and the null model A for branch *f* (2ΔlnL = 9.92, df = 1, *P* < 0.01) (Table 3), which suggested that 4.5% of the amino acids are under positive selection in lineage *f* with *ω* = ∞ (Table 1). Model A using branch *a* as the foreground branch (MAa) did not fit the data better than the two null models in test 1 and test 2 (Table 3). These evidences are sufficient to support the positive selection hypothesis on lineages *c*, *d,* and *f*.

**Table 3:** Likelihood ratio statistics (2ΔlnL) for testing branch-site hypothesis

	M1a	Branch-site MAa (*ω*_*a*_ = 1)	Branch-site MAc (*ω*_*c*_ = 1)	Branch-site MAd (*ω*_*e*_ = 1)	Branch-site MAf (*ω*_*e*_ = 1)
MAa	0.98 (0.61)	0.24 (0.89)			
MAc	25.56 (2.82E-07)**		11.58 (6.67E-04)**		
MAd	17.2 (1.84E-04)**			6.58 (1.03E-02)*	
MAf	31.82 (1.23E-07)**				9.92 (7.01E-03)**

* Significant (*P* < 0.05).

** Extremely significant (*P* < 0.01).

**Figure 2:**
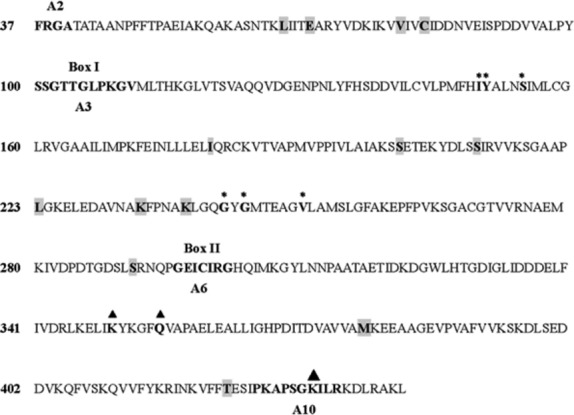
The deducted amino acid sequence for Arabidopsis At4CL1 referred to in this article. The residues involved in hydroxycinnamate binding are indicated with stars, while those involved in enzymatic functions are labeled with triangles (Hu et al. 2010). The bold-type letters indicate conserved motifs (Gulick 2009), while those on a gray background indicate positively selected sites.

Based on the BEB method, four and six candidate sites for positive selection were identified in dicots and monocots, respectively (Table 1). These positively selected sites are labeled in Figure2. Sites 181I, 202S, 211S, 223L, 234K, and 239K are located in the substrate-binding pocket, and 379M and 423T are located in the in catalytic centers. Sites 65L, 69E, 79V, and 82C are located between the conserved sequence motifs A2 and A3, which form a phosphate-binding loop. Site 291S is close to motif A6, which is important for the formation of a stable tertiary structure. Thus, amino acid substitutions in these positively selected sites in the 4CL genes might influence the 4CL substrate specificity, activity, or secondary structure, which would in turn have a profound effect on 4CL's function.

We have demonstrated that *4CL* genes in branches *c* and *f*, which are associated with lignin accumulation, are under positive selection. Interestingly, positive selection is also detected at the *At4CL* genes in branch *d*. However, the role of these proteins in lignin formation is similar to other proteins from dicots class I (Hu et al. 1998; Ehlting et al. 1999). We hypothesize that positive selection on the *At4CL* genes may be related to functional specialization.

### Selective constraints on flavonoid-related 4CLs in dicots

The *4CL* genes involved in flavonoid biosynthesis (dicots class II and monocots class II, Fig. 1) have been largely conserved during plant evolution, suggesting that they are constrained by purifying selection. Land plants evolved from green algae in the mid-Ordovician over 450 million years ago (Langdale 2008). After arriving in terrestrial environments, the pioneering land plants were confronted with several major challenges such as ultraviolet irradiation, desiccation stress. The presence of flavonoid in the earliest land plants and the associated ability to resist UV irradiations made survival on land possible for the plants (Rozemaa et al. 2002). Flavonoid evolved prior to the lignin pathway. For example, bryophytes do not synthesize lignin, but accumulate soluble phenylpropanoids, such as flavonoids and lignans (Weng and Chapple 2010). Flavonoids accumulate in the epidermal layer of extant plants, which has been shown to absorb over 90% of UV-B radiation (Stafford 1991). These evidences suggested that the ancestral role of 4CL was to participate in the flavonoid biosynthesis and that this role was maintained in the adaption to a terrestrial environment.

## Conclusions

4CLs play important roles in both lignin and flavonoid biosynthesis. 4CLs that play a role in lignin biosynthesis are subject to positive selection. This positive selection resulted in a functional divergence after the monocot–dicot split approximately 200 million years ago. Positive selection could have been involved in the early stages of the evolution of the *4CL* genes; *4CL* rapidly evolves after speciation events. Strong purifying selection operates on the novel *4CL* genes to maintain the protein's existing function. Based on the BEB method, four and six candidate sites for positive selection were identified in dicots and monocots, respectively (Table 1). Most of the positively selected sites are located in the substrate-binding pocket and the catalytic centers (Fig. 2). Therefore, amino acid replacements in these sites might imply a neofunctionalization. The result is in agreement with our findings that *4CL* genes functionally diversified in angiosperms (Hu et al. 1998; Ehlting et al. 1999; Gui et al. 2011; Xu et al. 2011; Sun et al. 2013). Although several positively selected sites were detected using the branch-site model, we find that the *4CL* gene family as a whole experiences purifying evolution rather than pervasive selection throughout evolution. The 4CLs involved in flavonoid biosynthesis have been largely conserved during plant evolution and maintain the ancestral role in response to biotic or abiotic factors. These findings provide deeper insights into understanding the evolutionary mechanisms of 4CL isoforms and their functional diversification.

## Supporting Information

**Table S1**. Members of plant 4-coumarate:coenzyme A ligase (*4CL*) genes.

**Table S2.** Likelihood Ratio Statistics (2ΔlnL) for Testing Method1 result.
