# Supplementary material for: Positive selection drives adaptive diversification of the 4-coumarate: CoA ligase (4CL) gene in angiosperms
Source: Ecol Evol. 2015 Jul 23;5(16):3413–20. doi: 10.1002/ece3.1613 (PMC4569036; doi:10.1002/ece3.1613)
Supplement: Supplementary file 1 — Table S1. Members of plant 4-coumarate:coenzyme A ligase (4CL) genes. [file ece30005-3413-sd1.doc]

**Table S1.  Members of plant 4-coumarate:coenzyme A ligase (*4CL*) genes**

| **Species** | **Gene name** | **Genebank Accession No.** |
| --- | --- | --- |
| ***Arabidopsis thaliana*** | *At4CL1* | U18675 |
|  | *At4CL2* | AF106086 |
|  | *At4CL3* | AF106088 |
|  | *At4CL4* | AAM19949 |
| ***Giycine max*** | *Gm4CL1* | AF279267 |
|  | *Gm4CL2* | AF002259 |
|  | *Gm4CL3* | AF002258 |
|  | *Gm4CL4* | X69955 |
| ***Gossypium hirsutum*** | *Gh4CL1* | FJ479707 |
|  | *Gh4CL2* | FJ848870 |
| ***Lithospermum erythrorhizon*** | *Le4CL1* | D49366 |
|  | *Le4CL2* | D49367 |
| ***Lolium perenne*** | *Lp4CL1* | AAF37732 |
|  | *Lp4CL2* | AAF37733 |
|  | *Lp4CL3* | AAF37734 |
| ***Nicotiana tabacum*** | *Nt4CL* | D43773 |
|  | *Nt4CL1* | U50845 |
|  | *Nt4CL2* | U50846 |
| ***Oryza Sativa*** | *Os4CL1* | NM001067888.1 |
|  | *Os4CL2* | NM001054354.1 |
|  | *Os4CL3* | NM001052604.1 |
|  | *Os4CL4* | NM001064787.1 |
|  | *Os4CL5* | NM001061935.1 |
| ***Panicum virgatum*** | *Pv4CL1* | EU491511.1 |
|  | *Pv4CL2* | JF414903 |
| ***Petroselinum crispum*** | *Pc4CL1* | X13324 |
|  | *Pc4CL2* | X13325 |
| ***Populus hybrida*** | *Poph4CL1* | AF008184 |
|  | *Poph4CL2* | AF008183 |
| ***Populus tremuloides*** | *Pt4CL1* | AF041049 |
|  | *Pt4CL2* | AF041050 |
| **Loblolly pine** | *Lp4CL1* | U12012 |
|  | *Lp4CL2* | U12013 |
| ***Rubus idaeus*** | *Ri4CL1* | AF239687 |
|  | *Ri4CL2* | AF239686 |
|  | *Ri4CL3* | AF239685 |
| ***Salvia miltiorrhiza*** | *Sm4CL1* | AY237163 |
|  | *Sm4CL2* | AY237164 |
| ***Solanum tuberosum*** | *St4CL1* | M62755 |
|  | *St4CL2* | AF150686 |
| ***Sorghum bicolor*** | *Sb4CL* | AAA64913.1 |
|  | *Sb4CL2* | XP-002451647.1 |
