# Supplementary material for: Positive selection drives adaptive diversification of the 4-coumarate: CoA ligase (4CL) gene in angiosperms
Source: Ecol Evol. 2015 Jul 23;5(16):3413–20. doi: 10.1002/ece3.1613 (PMC4569036; doi:10.1002/ece3.1613)
Supplement: Supplementary file 2 — Table S2. Likelihood Ratio Statistics (2ΔlnL) for Testing Method1 result. [file ece30005-3413-sd2.doc]

**Table S2. Likelihood Ratio Statistics (2ΔlnL) for Testing Method1 result**

| **Method compared** | **P value** |  | **Method compared** | **P value** |
| --- | --- | --- | --- | --- |
| TRM vs ORM | **0.00E+00** |  | 41-RMvs40-RM | 5.39E-01 |
| 3-RM vs TRM-22 | **1.18E-06** |  | 42-RM vs 40-RM | 3.64E-01 |
| 4-RM vs 3-RM | **3.26E-08** |  | 43-RM vs 40-RM | 5.42E-01 |
| 5-RM vs 4-RM | **4.17E-10** |  | 44-RM vs 40-RM | 4.74E-01 |
| 6-RM vs 5-RM | **1.74E-08** |  | 45-RM vs 40-RM | 4.62E-01 |
| 7-RM vs 6-RM | **4.69E-06** |  | 46-RM vs 40-RM | 4.47E-01 |
| 8-RM vs 7-RM | **8.96E-06** |  | 47-RM vs 40-RM | 4.99E-01 |
| 9-RM vs 8-RM | **1.99E-03** |  | 48-RM vs 40-RM | 6.00E-01 |
| 10-RM vs 9-RM | **6.31E-04** |  | 49-RM vs 40-RM | 3.63E-01 |
| 11-RM vs 10-RM | **2.19E-04** |  | 50-RM vs 40-RM | 4.38E-01 |
| 12-RM vs 11-RM | **3.66E-02** |  | 51-RM vs 40-RM | 4.89E-01 |
| 13-RM vs 12-RM | **4.74E-04** |  | 52-RM vs 40-RM | 4.70E-01 |
| 14-RM vs 13-RM | **4.22E-04** |  | 53-RM vs 40-RM | 5.13E-01 |
| 15-RM vs 14-RM | **3.53E-07** |  | 54-RM vs 40-RM | 3.25E-01 |
| 16-RM vs 15-RM | **2.18E-01** |  | 55-RM vs 40-RM | 9.06E-02 |
| 17-RM vs 15-RM | **2.80E-03** |  | 56-RM vs 40-RM | 9.67E-02 |
| 18-RM vs 17-RM | **8.38E-04** |  | 57-RM vs 40-RM | 1.11E-01 |
| 19-RM vs 18-RM | **2.83E-02** |  | 58-RM vs 40-RM | 1.48E-01 |
| 20-RM vs 19-RM | **2.04E-03** |  | 59-RM vs 40-RM | 1.85E-01 |
| 21-RM vs 20-RM | **3.68E-04** |  | 60-RM vs 40-RM | 2.18E-01 |
| 22-RM vs 21-RM | **3.73E-06** |  | 61-RM vs 40-RM | 2.28E-01 |
| 23-RM vs 22-RM | **1.41E-02** |  | 62-RM vs 40-RM | 2.73E-01 |
| 24-RM vs 23-RM | 5.12E-02 |  | 63-RM vs 40-RM | 2.38E-01 |
| 25-RM vs 23-RM | **6.40E-04** |  | 64-RM vs 40-RM | 2.84E-01 |
| 26-RM vs 25-RM | **6.81E-03** |  | 65-RM vs 40-RM | 3.15E-01 |
| 27-RM vs 26-RM | **6.38E-03** |  | 66-RM vs 40-RM | 3.44E-01 |
| 28-RM vs 27-RM | 3.10E-01 |  | 67-RM vs 40-RM | 2.40E-01 |
| 29-RM vs 27-RM | 9.84E-02 |  | 68-RM vs 40-RM | 2.77E-01 |
| 30-RM vs 27-RM | **3.79E-02** |  | 69-RM vs 40-RM | 3.21E-01 |
| 31-RM vs 30-RM | 8.49E-02 |  | 70-RM vs 40-RM | 3.32E-01 |
| 32-RM vs 30-RM | **4.94E-02** |  | 71-RM vs 40-RM | 3.72E-01 |
| 33-RM vs 32-RM | **1.58E-02** |  | 72-RM vs 40-RM | 3.82E-01 |
| 34-RM vs 33-RM | 1.45E-01 |  | 73-RM vs 40-RM | 4.31E-01 |
| 35-RM vs 33-RM | 8.72E-02 |  | 74-RM vs 40-RM | 4.80E-01 |
| 36-RM vs 33-RM | 9.00E-02 |  | 75-RM vs 40-RM | 5.23E-01 |
| 37-RM vs 33-RM | 1.64E-01 |  | 76-RM vs 40-RM | 5.63E-01 |
| 38-RM vs 33-RM | 1.70E-01 |  | 77-RM vs 40-RM | 6.09E-01 |
| 39-RM vs 33-RM | 1.99E-01 |  | 78-RM vs 40-RM | 6.01E-01 |
| 40-RM vs 33-RM | **3.89E-02** |  | 79-RM vs 40-RM | 6.45E-01 |

Significant P values are shown in bold
